# Optimization of Ultrasonic-Assisted Extraction of Cordycepin from *Cordyceps militaris* Using Orthogonal Experimental Design

**DOI:** 10.3390/molecules191220808

**Published:** 2014-12-12

**Authors:** Hsiu-Ju Wang, Meng-Chun Pan, Chao-Kai Chang, Shu-Wei Chang, Chang-Wei Hsieh

**Affiliations:** 1 Department of Hospitality Management, Mingdao University, 369 Wen-Hua Rd, Peetow, Chang-Hua 52345, Taiwan; E-Mail: hjwang@mdu.edu.tw; 2 Department of Medicinal Botanicals and Health Applications, Da-Yen University, 168 University Rd, Dacun, Chang-Hua 51591, Taiwan; E-Mails: juichen0812@gmail.com (M.-C.P.); swchang@mail.dyu.edu.tw (S.-W.C.); 3 Department of BioIndustry Technology, Da-Yeh University, 168 University Rd, Dacun, Chang-Hua 51591, Taiwan; E-Mail: kai0913077636@gmail.com

**Keywords:** ultrasonic-assisted extraction, *Cordyceps militaris*, cordycepin, orthogonal experimental design

## Abstract

This study reports on the optimization of the extraction conditions of cordycepin from *Cordyceps militaris* by using ultrasonication. For this purpose, the orthogonal experimental design was used to investigate the effects of factors on the ultrasonic-assisted extraction (UAE). Four factors: extraction time (min), ethanol concentration (%), extraction temperature (°C) and extraction frequency (kHz), were studied. The results showed that the highest cordycepin yield of 7.04 mg/g (86.98% ± 0.23%) was obtained with an extraction time of 60 min, ethanol concentration of 50%, extraction temperature of 65 °C and extraction frequency of 56 kHz. It was found that the cordycepin extraction yield increased with the effect of ultrasonication during the extraction process. Therefore, UAE can be used as an alternative to conventional immersion extraction with respect to the recovery of cordycepin from *C. militaris*, with the advantages of shorter extraction time and reduced solvent consumption.

## 1. Introduction

**Figure 1:**
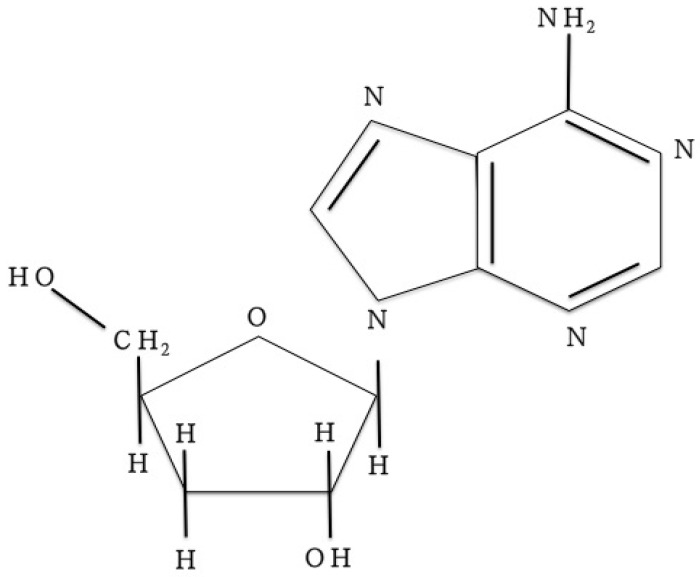
Chemical structures of cordycepin [15].

*Cordyceps militaris*, which is one of the medicinal mushrooms, is an entomopathogenic fungi belonging to family Clavicipitaceae and Ascomycotina [1]. It has similar pharmacological activitiesto the well-known Chinese traditional medicine *Cordyceps sinensis* [2]. Besides its usage as a crude drug, it has been extensively used as folk tonic food or an invigorant since ancient times [3,4]. The major bioactive compound of *C. militaris* is cordycepin (3’-deoxyadenosine), which is a nucleoside analogue [3,5]. Cordycepin was first isolated from a culture broth of *C. militaris* in the 1950s [6]. Since then, many biological and pharmacological functions of cordycepin, including anti-virus, anti-cancer, anti-diabetic, anti-inflammatory, renoprotective and immunomodulatory activities have been discovered [1,5,6,7,8,9,10,11,12,13]. In the last decade, cordycepin (Figure 1) has been studied as a therapeutic agent for a variety of cancers, especially leukemia (ClinicalTrials.gov, Verified by OncoVista, Inc., San Antonio, TX, USA, 2009), trypanosomiasis and restenosis [14]. These studies imply the large and increasing need for cordycepin.

Although cordycepin can be chemically synthesized, yields are low and the processes are complicated; extraction from fruiting bodies of *C. militaris* still remains its main source [16,17]. Cordycepin could be extracted from a fermentative solution and from fruiting bodies of *C. militaris* by several conventional methods, such as pressurized extraction, Soxhlet extraction and reflux extraction [18]. However, these are often inefficient, as well as solvent- and time-consuming. Ultrasonic-assisted extraction (UAE) is a novel method used to enhance production yield as well as to avoid thermal damage. UAE has been used in numerous studies to extract natural products [19] and improve solvent extraction, mainly due to the mechanical effects of cavitational bubble collapse, causing better solvent penetration into plant materials [20,21]. Therefore, UAE is a highly efficient method with reduced solvent- and time-consumption.

Esclapez *et al.* (2011) reported that several process variables: ultrasonic power, frequency, extraction temperature, reactor characteristics and solvent–sample interaction can influence the extraction [22,23]. Mathematical modeling is an effective statistical model it include theoretical and statistical model for investigating the influences of different factors on extraction, as well as the search for the optimal conditions [24]. Among the current modeling methodologies, orthogonal experimental design is a highly efficient way for dealing with multifactor experiments and screening optimum levels by using the orthogonal design table and statistical analysis [25]. Regressive analysis can be used to obtain the optimized parameters, in order to achieve the predetermined features and uncover the statistic principle based on the hidden or equivocal factors [26]. For example, for an experiment with four factors and four levels of each factor, an orthogonal design table L16(4^4^) could be used; the experiment program only contains 16 level groups, reflecting the overall situation of the comprehensive experiment containing 256 level groups in all. Thus, it is much easier to derive the optimum level group.

The aims of this study were to investigate the effect of ultrasonic on the extraction efficiency of cordycepin from *C. militaris*, as well as to optimize the parameters of this process by orthogonal experimental design.

## 2. Results and Discussion

### 2.1. Effects of Extraction Variables on Extraction Yield of Cordycepin

#### 2.1.1. Extraction Time

**Figure 2:**
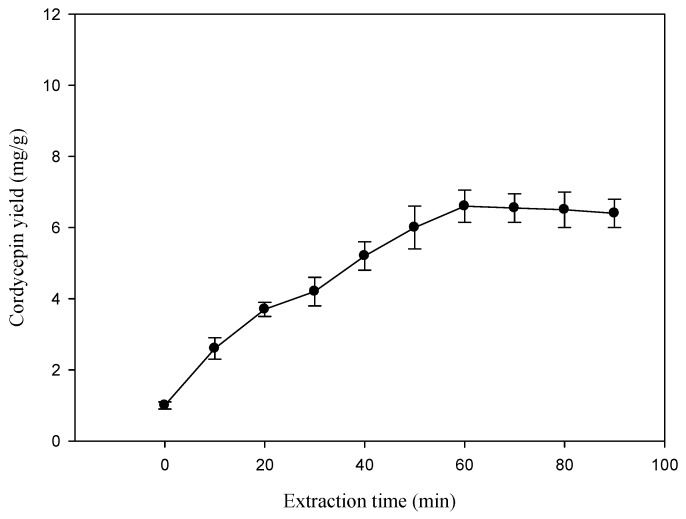
Effect of extraction time on the extraction yield of cordycepin from *C. militaris*. Ethanol concentration: 50%; extraction temperature: 60 °C; ratio of liquid to solid: 20 mL/g; extraction frequency: 40 kHz.

The effects of the extraction time on the extraction yield of cordycepin from *C. militaris* were investigated; the other experimental parameters were 50% ethanol (in water) with a ratio of liquid to material at 20:1, and extracting temperature at 60 °C under ultrasonic irradiation. The results are shown in Figure 2. The yields significantly increased when the extraction time increased from 0 to 60 min, and then the yields were almost unchanged from 60 to 90 min. A longer extraction time indicated a positive effect on the extraction yield, but the yield increased slightly. This phenomenon may be due to the active ingredients will not be dissolved when the solubility of dissolving-out substances became saturated with the increase of extraction time, while the loss of cordycepin were increased with the viscosity of extracts increased when extraction time increased [27]. Therefore, in view of time consumption, extraction time ought not to exceed 70 min.

#### 2.1.2. Ethanol Concentration

**Figure 3:**
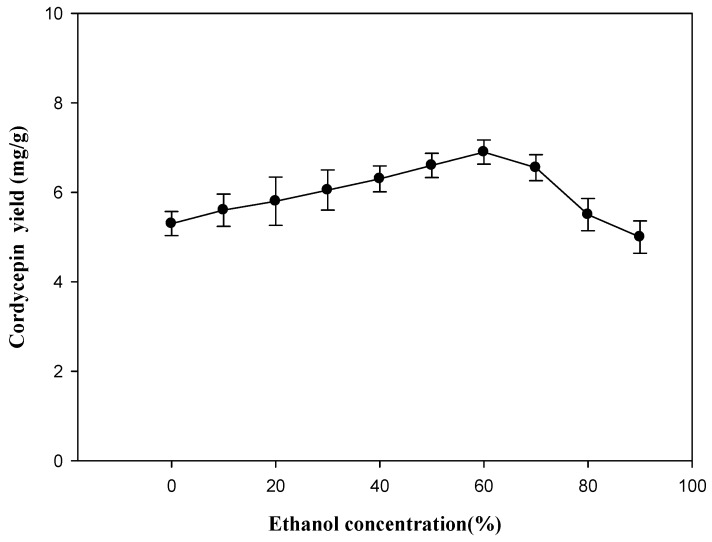
Effect of ethanol concentration on the extraction yield of cordycepin from *C. militaris*. Extraction time: 50 min; extraction temperature: 60 °C; ratio of liquid to solid: 20 mL/g; extraction frequency: 40 kHz.

*C. militaris* was used as the material for extraction condition optimization. Based on literature reports [28] and our pre-experiment (data not shown), water, methanol and ethanol were adopted as the extraction solvents; the results show that the extraction efficiency of ethanol is best on the grounds of safety and efficiency. Hence, ethanol was chosen as the best solvent in the following extraction experiments. The impact of ethanol concentration on the extraction yield of cordycepin was demonstrated, and is shown in Figure 3. The extraction yield increases first when the ethanol concentration changes from 0% to 60% (*v*/*v*), and goes down when the ethanol concentration is above 70%. The extraction process is usually controlled by the solubility of the solute. Moreover these results supported previous findings that ethanol/water was the best solvent for the extraction of cordycepin from different natural products [28]. Therefore, the preferred ethanol concentration is 60%, as the maximum extraction yield is, thus, obtained.

#### 2.1.3. Extraction Temperature

**Figure 4:**
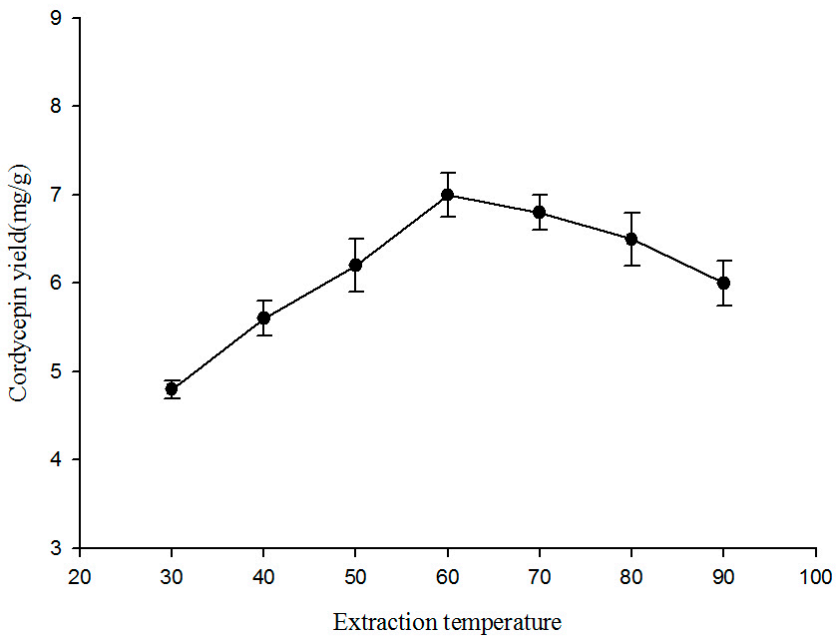
Effect of extraction temperature (°C) on the extraction yield of cordycepin from *C. militaris*. Extraction time: 50 min; ethanol concentration: 50%; ratio of liquid to solid: 20 mL/g; extraction frequency: 40 kHz.

Figure 4 shows that the extraction yield of cordycepin rises as the extraction temperature increases from 30 to 60 °C, and goes down when the temperature is above 70 °C. The extraction temperature could markedly influence the recovery of bioactive ingredients during liquid–solid extraction [29]. Increasing the temperature of the extraction medium can increase the diffusivity of the solvent into the cells; it can also enhance desorption and solubility of the target compounds of the cells, resulting in the dissolution of the components [30,31] However, when the extraction temperature goes beyond a certain threshold (60 to 70 °C), the extraction yield starts to decrease. This may be ascribed to the decreased number of acoustic cavitation bubbles created by the ultrasound and the thermal degradation of cordycepin. Additionally, increasing extraction temperature might result in accelerated solvent volatilization, improved energy cost and enhanced extraction of impurities [32].

#### 2.1.4. Ratio of Liquid to Solid

**Figure 5:**
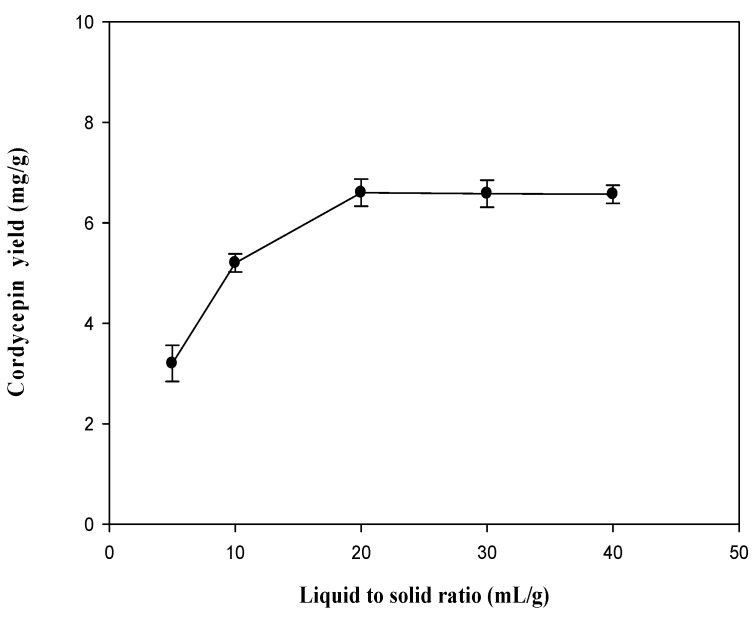
Effect of ratio of liquid to solid on the extraction yield of cordycepin from *C. militaris*. Extraction time: 50 min; ethanol concentration: 50%; extraction temperature: 60 °C; extraction frequency: 40 kHz.

Generally, the large solvent volume dissolves target components more effectively, and results in an enhancement of extraction yield [30,33,34]. Figure 5 shows the influence of the ratio of liquid to solid on the extraction of cordycepin. The result indicates that the yields significantly increased when the ratio of liquid to solid increased from 5 to 20 mL/g; after 20 mL/g, the yield of cordycepin was almost unchanged. Therefore, a volume of 20 mL was sufficient for extraction.

#### 2.1.5. Extraction Frequency

**Figure 6:**
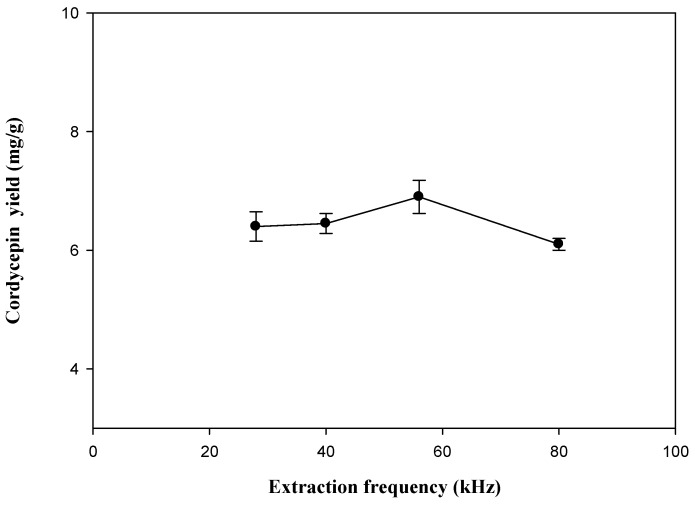
Effect of extraction frequency on the extraction yield of cordycepin from *C. militaris*. Extraction time: 50 min; ethanol concentration: 50%; extraction temperature: 60 °C; ratio of liquid to solid: 20 mL/g.

The effects of extraction frequency on the extraction yield of cordycepin from *C. militaris* were investigated; the other experimental parameters were 50% ethanol with a ratio of liquid to material at 20:1, and extracting temperature at 60 °C under ultrasonic irradiation. The results are shown in Figure 6. As we can see, the maximum yield could be obtained at 56 kHz, which was 6.89 mg/g. When a higher frequency ultrasound is employed, the extraction yield did not increase significantly. This result was in agreement with the past findings [27].

### 2.2. Final Optimization by Orthogonal Experimental Design

Since various parameters potentially affect the UAE process, the optimization of the experimental conditions represents a critical step in the development of a UAE method. An orthogonal test of UAE of cordycepin was designed based on the previous results from single factor experiments in order to optimize the combination of parameters. Four factors, extraction time, ethanol concentration, extraction temperature and extraction frequency, were selected for optimization (Table 1). For four factors at four levels each, the orthogonal test design required only 16 experiments while the traditional full factorial design would require 44 or 256 experiment. 

**Table 1:** Factors and levels of orthogonal experimental design.

Levels	Factors			
	A	B	C	D
	Extraction Time (min)	Ethanol Concentration (%)	Extraction Temperature (°C)	Extraction Frequency (kHz)
1	40	30	55	28
2	50	40	60	40
3	60	50	65	56
4	70	60	70	80

The analysis of extreme difference indicates that the influential order of the four factors on the extraction yield of cordycepin is extraction time > ethanol concentration > extraction temperature > extraction frequency (Table 2). The order is in agreement with the order based on the values of F in variance analysis (Table 3). According to variance analysis, the contributions of extraction time (*p* < 0.01) and ethanol concentration (*p* < 0.05) for the extraction yield of cordycepin are significant, whereas extraction temperature and extraction frequency are not significant factors. According to extreme difference analysis, the optimum extraction condition of cordycepin was deduced as: extraction time of 60 min, ethanol concentration of 50%, extraction temperature of 65 °C and extraction frequency of 56 kHz. Unfortunately, this combination was not included in the orthogonal test. Fortunately, extraction time and ethanol concentration, which significantly affect the extraction yield of cordycepin, are at the optimum levels in the eleventh experiment of orthogonal test. The higher extraction yield (83.16%) in the eleventh experiment provided evidence that the deduced extraction condition might be the optimum one. To reconfirm this deduced optimum condition, UAE under this condition was carried out, and the extraction yield of cordycepin reached 7.04 mg/g (86.98% ± 0.23%). So this deduced condition was rationally confirmed to be the best combination of different parameters.

**Table 2:** Orthogonal test design and results.

EXP	Factors				Extraction Yield (%)
	A	B	C	D	
1	1	1	1	1	60.03 ± 0.62
2	1	2	2	2	70.84 ± 0.56
3	1	3	3	3	75.41 ± 0.63
4	1	4	4	4	74.53 ± 0.57
5	2	1	2	3	80.67 ± 0.47
6	2	2	1	4	77.93 ± 0.31
7	2	3	4	1	82.23 ± 0.24
8	2	4	3	2	81.43 ± 0.44
9	3	1	3	4	78.01 ± 0.63
10	3	2	4	3	77.84 ± 0.52
11	3	3	1	2	83.16 ± 0.54
12	3	4	2	1	84.02 ± 0.33
13	4	1	4	2	71.68 ± 0.65
14	4	2	3	1	76.24 ± 0.23
15	4	3	2	4	80.08 ± 0.34
16	4	4	1	3	80.23 ± 0.44
K1	280.81	290.39	290.39	302.52	-
K2	322.26	302.85	302.85	307.11	-
K3	323.03	320.88	311.09	314.15	-
K4	308.23	320.21	306.28	310.55	-
k1	70.20	72.60	72.60	75.63	-
k2	80.57	75.71	75.71	76.78	-
k3	80.76	80.22	77.77	78.54	-
k4	77.06	80.05	76.57	77.64	-
R	10.56	7.62	5.18	2.91	-

**Table 3:** Variance analysis of orthogonal test.

Factors	SS	df	MS	F	P	Significant
A	292	3	97.270020	9.080411	0.051436	**
B	163	3	54.187990	5.058590	0.108024	*
C	28	3	9.440320	0.881279	0.540148	-
D	18	3	6.149190	0.574043	0.670151	-
Error	32.1362	51	10.71207	-	-	-
Total	9.31	26	-	-	-	-

F_0.01_ (3,51) = 5.28 F_0.05_ (3,51) = 2.79; SS: Sum of square; df: Degree of freedom; MS: Mean of square; F: F-value; **: *p* < 0.01 (F > F_0.0__1_ (3,51)); *: *p* < 0.05 (F > F_0.05_ (3,51)); A: Extraction time; B: Ethanol concentration; C: Extraction temperature; D: Extraction frequency.

### 2.3. Comparison between UAE and Conventional Immersion Extraction

Table 4 indicates the comparison of the results obtained by UAE and conventional immersion extraction. After 1 h extraction, the recovery by UAE at extraction temperature of 65 °C with 60 mL of 50% ethanol is approximately 4.1 times higher than that by conventional immersion extraction at 65 °C with 750 mL of 50% ethanol (86.98% *vs.* 21.43%). Conventional immersion extraction could not produce the same level of recovery (95.23%) until after 24 h. considering cost and time consumption, UAE is much more economical in terms of time, solvent and energy. The high efficiency of UAE found in this work was suggested to be because the cells of *C. militaris* were broken by the ultrasound, so that cordycepin dissolved more easily in the solvent. Therefore, UAE was the optimal method for extracting cordycepin.

**Table 4:** Comparison of UAE and conventional immersion extraction.

Extraction Method	Ultrasonic-Assisted Extraction	Immersion Extraction	
Extraction time (h)	1	1	24
Extraction temperature (°C)	65	65	65
Ratio of liquid to solid (mL/g)	1:20	1:250	1:250
Recovery (%)	86.98% ± 0.23% ^b^	21.43% ± 1.13% ^c^	95.23% ± 3.11% ^a^

Means of replicates ± S.E. (N = 3); a–c: The means in the same column followed by different letters are significantly different at *p* < 0.05.

## 3. Experimental Section

### 3.1. Materials

Cordycepin was purchased from Sigma (St. Louis, MO, USA). An amount of 95 g/100 g ethanol was purchased from Taiwan Tobacco Co. (Taipei, Taiwan). All of the other chemicals used in this study were of analytical grade and were obtained commercially.

Fresh fruiting bodies of *C. militaris* grown on cereals were obtained from Wah-Lee Biotech Co. (Changhua, Taiwan). The fruiting bodies were washed with deionized water, freeze-dried, crushed by the homogenizer, and finally kept in the desiccator in properly sealed containers until extraction.

### 3.2. Conventional Immersion Extraction

In order to compare the extraction ability of the UAE technique and conventional solvent extraction (immersion), the immersion was carried out after optimizing the UAE process. Three grams of *C. militaris* fruiting bodies samples mixed with 750 mL of 50% (*v*/*v*) ethanol were placed in a water bath (65 °C) for 1 and 24 h, respectively. After the extraction process was completed, the filtrate and the retentate were separated by suction filtration. The filtrate was then filtered by a 0.45 μm filter for further experiments.

### 3.3. Ultrasonic-Assisted Extraction

In UAE, the ultrasound cleansing tank has four frequencies, 28, 40, 56 and 80 kHz, with temperature- and time-controlling panels (Tex-Ray Industrial Co., Changhua, Taiwan). Each of the 3.0 g of *C. militaris* fruiting bodies sample was mixed with 60 mL of 50% ethanol, and the mixture was directly sonicated in the ultrasonic extraction tank for 60 min at 65 °C and at a frequency of 56 kHz. After extraction, suction filtration (by 0.45 μm membranes) was used to obtain the filtrate.

### 3.4. Optimization of Ultrasonic-Assisted Extraction Condition

Firstly, the effects of ethanol concentration (0% to 90% *v*/*v*), extraction time (0 to 90 min), extraction temperature (30 to 90 °C), ratio of liquid to solid (5 to 40 mL/g) and extraction frequency (28, 40, 56 and 80 kHz) on the extraction yield of cordycepin from *C. militaris* were investigated. Secondly, an orthogonal test was designed to optimize the extraction parameters (factors) depending on the results of the above single factor experiments. The factors and levels tested in this study are presented in Table 1. The orthogonal test design consisted of sixteen separate experiments (Table 2). The sequence in which the experiments were performed was randomized to ensure the validity of the test results. In this study, all the experiments were performed in triplicate.

### 3.5. Analysis of Cordycepin in the Extracts

The cordycepin contents of all the samples were determined by the method of Ni *et al.* (2009) [18] with minor modifications. The cordycepin contents were analyzed on an RP-18 column (250 mm × 4.6 mm, 5 μm) by a Hitachi L-7400 HPLC system (Tokyo, Japan). The flow rate was 1 mL/min, the composition ratio of the mobile phase was water: methanol = 85: 15 (*v*/*v*) and the injection amount was 20 μL. Cordycepin were detected at 260 nm. The samples were filtered through a 0.45 μm membrane filter before injection. The extraction yield of cordycepin (mg·g^−1^) was calculated as the amount of the extracted cordycepin (mg) per gram of fruiting bodies samples. Extraction yield (%) = (the amount of cordycepin in extract/sample mass) × 100.

## 4. Conclusions

An efficient UAE was employed to extract cordycepin from *C. militaris.* An orthogonal experimental design was used to determine the optimum extraction parameters that give high extraction yield. It was found that the optimum extraction condition of cordycepin was extraction time of 60 min, ethanol concentration of 50%, extraction temperature of 65 °C and extraction frequency of 56 kHz. Of the four extraction factors, extraction time and ethanol concentration have significant effects on the extraction yield of cordycepin based on statistical analysis. The highest cordycepin extraction yield, 7.04 mg/g (86.98% ± 0.23%), was obtained by using the orthogonal experimental design optimization. Compared with conventional immersion extraction, UAE can shorten extraction time and decrease solvent consumption. Therefore, UAE shows strong potential as a method for enhancing cordycepin concentration during extraction from *C. militaris.*
